# Effect of Avocado Consumption on Risk Factors of Cardiovascular Diseases: A Systematic Review and Meta-Analysis

**DOI:** 10.7759/cureus.41189

**Published:** 2023-06-30

**Authors:** Okelue E Okobi, Victor A Odoma, Omolola Okunromade, Olusayo Louise-Oluwasanmi, Blessing Itua, Chinonso Ndubuisi, Omosefe E Ogbeifun, Bright C Nwatamole, Thomas A Elimihele, Joy O Adekunle, Akeem A Adekunle, Chinedum B Obi, Endurance O Evbayekha

**Affiliations:** 1 Family Medicine, Larkin Community Hospital, Miami, USA; 2 Family Medicine, Lakeside Medical Center, Belle Glade, USA; 3 Cardiology/Oncology, Indiana University (IU) Health Bloomington Hospital, Bloomington, USA; 4 Public Health, Jiann-Ping Hsu College of Public Health, Georgia Southern University, Statesboro, USA; 5 Genetics, Howard University College of Medicine, Washington, USA; 6 Internal Medicine, Annotto Bay Hospital, St. Mary, JAM; 7 Family Medicine, Humboldt Park Health, Chicago, USA; 8 Public Health, University of West Florida, Florida, USA; 9 Cardiology, Calderdale and Huddersfield NHS Foundation Trust, Huddersfield , GBR; 10 Clinical Research, Norris Comprehensive Cancer Center, University of Southern California, Los Angeles, USA; 11 Internal Medicine, Lagos State Health Service (LHSC), Lagos, NGA; 12 Internal Medicine, College of Medicine, University of Lagos, Lagos, NGA; 13 Internal Medicine, Imo State University, Owerri, NGA; 14 Internal Medicine, St. Luke's Hospital, Chesterfield, USA

**Keywords:** avocado, cardiovascular disease, avocado diet, risk factors for heart disease, low fat diets, total cholesterol

## Abstract

High cholesterol levels are a significant risk factor for heart disease, the leading cause of death worldwide. Lowering cholesterol plays a crucial role in maintaining good health. One approach to reducing cholesterol is through dietary modifications, and avocados have been recognized as a potential food choice for this purpose. Avocados are rich in monounsaturated fatty acids (MUFAs), fiber, and plant sterols, which have cholesterol-lowering effects. Incorporating avocados into a low-fat diet can be beneficial. This study design followed the Preferred Reporting Items for Systematic Reviews and Meta-Analysis (PRISMA) guidelines and conducted databases in Cochrane, SCOPUS, PubMed, and Web of Science up until May 2023, combining keywords related to avocados and cardiovascular diseases (CVDs). The study focused on randomized clinical trials (RCTs) and excluded observational studies, meta-analyses, surveys, abstracts, and reviews. Seven RCTs were included in the study, all reporting total cholesterol (TC) levels. The findings of the study showed that individuals who followed an avocado diet experienced reduced TC levels compared to those who followed a habitual diet or a low-fat diet. The avocado group exhibited lower TC levels compared to the control group in both the habitual diet and low-fat diet subgroups. When considering high-density lipoprotein (HDL) levels, the control group had higher HDL levels than the avocado group in the habitual diet subgroup, while the avocado group had higher HDL levels than the control group in the low-fat diet subgroup. In both the habitual diet and low-fat diet subgroups, the avocado group had lower levels of low-density lipoprotein (LDL) compared to the control group. The study concluded that incorporating avocados into the diet can be a beneficial dietary strategy for individuals aiming to lower their cholesterol levels and promote heart health. The avocado diet was associated with decreased LDL levels, but it did not significantly impact triglyceride (TG) levels or fasting glucose levels. Systolic blood pressure values showed minimal changes with the avocado diet.

## Introduction and background

According to global estimates, poor diet is responsible for 255 million disability-adjusted life years and 11 million deaths [1]. Higher intakes of refined carbohydrates, salt, and saturated fat, as well as lower intakes of fiber-rich foods (vegetables, fruit, and whole grains), are consistently linked to cardiometabolic risk factors [1,2]. High blood cholesterol levels, particularly low-density lipoproteins (LDLs), are usually regarded as a significant risk factor for coronary heart disease (CHD) [3,4]. High levels of high-density lipoprotein (HDL), on the other hand, protect against the development of CHD [5-7]. To reduce blood cholesterol levels, many dietary regimens have been advised [3-5]. These suggestions generally include lowering overall fat and increasing fiber and polyunsaturated fatty acids in the diet. The oxidative alteration of LDL particles is crucial in the pathophysiology of atherosclerosis [8,9]. Oxidized LDL (oxLDL) is taken up by macrophages through the activated scavenger receptor, resulting in significant cholesterol buildup and the production of foam cells. Many longitudinal studies have demonstrated a high plasma oxLDL concentration as an independent risk factor for cardiovascular disease (CVD) [10,11]. Avocados consist of several numbers of phytochemicals and nutrients, and each of them plays a beneficial role in preventing CVD and improving the lipid profile [12]. Avocados contain ∼10% monounsaturated fatty acids (MUFAs). According to the American Heart Association guidelines, MUFAs are highly recommended instead of trans fatty acids and saturated fatty acids (SFAs) for the prevention of primary and secondary CVD [13]. When MUFAs replaced carbohydrates in the diet, the HDL cholesterol level showed elevations with decreasing in other lipid markers for CVD [14]. The higher MUFA intake was associated with an overall risk reduction in cardiovascular mortality (12%), all-cause mortality (11%), stroke (17%), and cardiovascular events (9%) than with the lower MUFA intake [15]. Avocados contain magnesium, potassium, and fiber, which are linked to cardiovascular health [12]. Avocado carbohydrates consist of∼80% dietary fiber, which is composed of 70% insoluble and 30% soluble fiber [16]. A recent meta-analysis showed that eating more fiber is associated with a decreased risk of CVD [17]. In our study, we aim to compare the efficacy of the avocado-rich diet with that of different avocado-free diets on CVD risk.

Materials and methods

Search Strategy and Information Sources

We conducted our study based on the Preferred Reporting Items for Systematic Reviews and Meta-Analysis (PRISMA) recommendations and guidelines [18]. We developed a search strategy by combining these keywords: (''Avocado'' OR ''Avocado-rich'' or ''Avocado-free'') AND (''cardiovascular diseases'' OR ''heart diseases''). Concerning data sources, we utilized Cochrane, SCOPUS, PubMed, and Web of Science databases till May 2023 for articles that matched our inclusion criteria in the search process.

Study selection

First, we performed both title and abstract screening. After that, we conducted a full-text screening. Finally, we selected eligible articles based on the following criteria:

· Population: The study included individuals who were either at an increased risk of CVD or were healthy.

· Intervention: The participants were provided with a diet rich in avocados.

· Comparator: The control group consisted of individuals who were given a diet without avocados.

· Outcomes: The outcomes assessed in the study were total cholesterol (TC) levels, triglyceride (TG) levels, LDL cholesterol levels, HDL cholesterol levels, fasting blood glucose (FBG) levels, systolic blood pressure (SBP), and diastolic blood pressure (DBP).

· Study design: We specifically included only randomized clinical trials (RCTs) and excluded observational studies, meta-analyses, surveys, abstracts, and reviews.

For quality assessment regarding the clinical trials, we utilized the Cochrane risk of bias tool, which depends on assessing eight domains in each clinical trial [19]. The tool assesses proper randomization of patients, allocation concealment, and adequate blinding through seven domains. Each domain could be categorized as high, unclear, or low risk of bias.

Data extraction

We extracted three types of data from included articles: the first category is the demographic characteristics of the involved individuals, the baseline, and the characteristics of the included trials. The second category was extracting data of the following outcomes for analysis: TC level, TG, LDL, HDL, FBG, SBP, and DBP. The last category was quality assessment data in both trials. The process of data collection was conducted using Microsoft Excel [20].

Data Synthesis and Analysis

We performed the meta-analysis of this study using Review Manager software (The Nordic Cochrane Center, Odense, Denmark) [21]. Our study included continuous outcomes. We analyzed continuous data using mean difference (MD) and 95% confidence interval (CI). The fixed-effects model was used when data were homogeneous, while heterogeneous data were analyzed under a random-effects model. To measure the presence of inconsistency among the studies, in 2 of 11 we used the *I*^2^ value and the *P*-value of the chi-square tests [22]. Values of *P* < 0.1 or *I*^2^ > 50% were significant indicators of the presence of heterogeneity. We tried to solve the inconsistency of heterogeneous outcomes using Cochrane's leave-one-out method [22].

Summary of Included Studies

**Figure 1:**
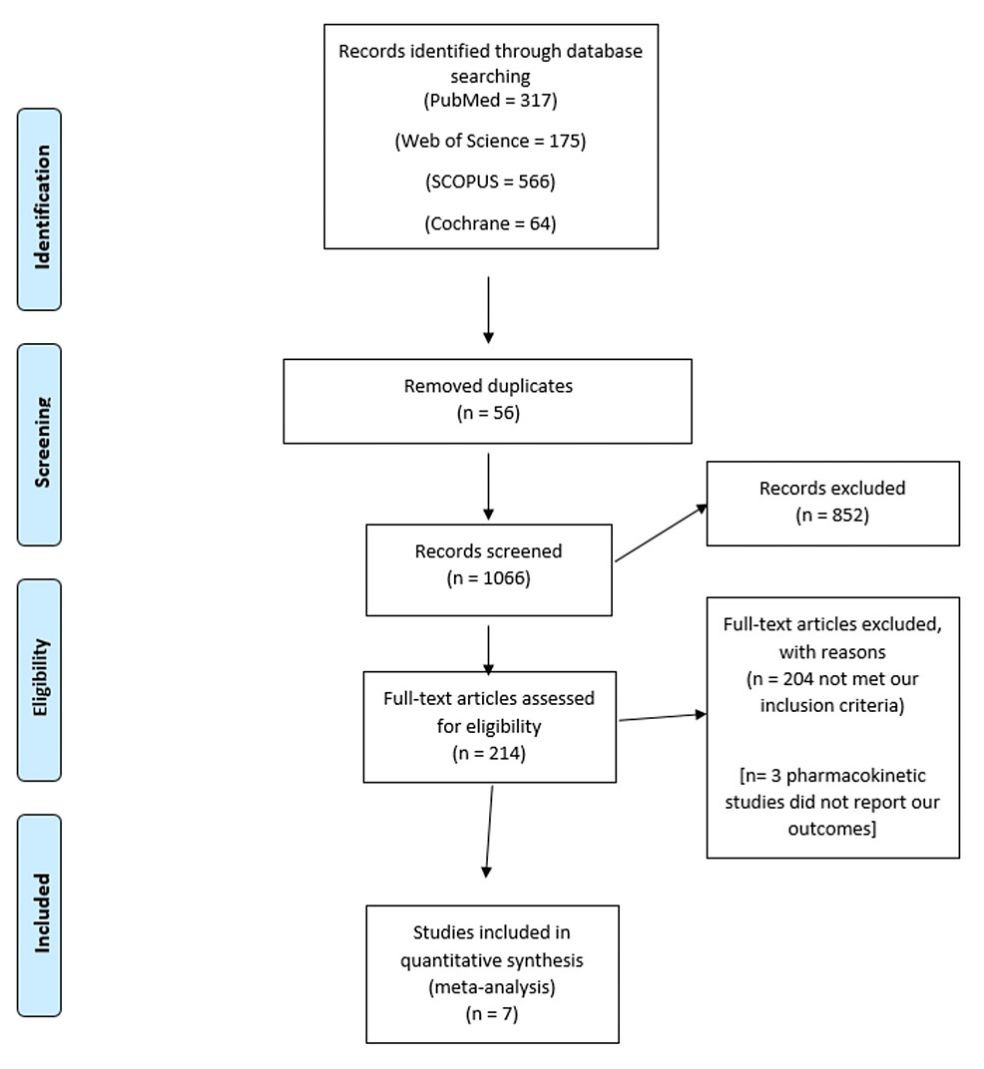
PRISMA flowchart of selected studies. PRISMA, Preferred Reporting Items for Systematic Reviews and Meta-Analysis

Figure 1 shows the PRISMA flowchart of our literature search process. A total of seven trials [23-29] were included in our meta-analysis. Our meta-analysis included 1,359 patients divided into two groups; the avocado group, which included 683 patients fed an avocado-rich diet, and the control group, which included 676 patients fed an avocado-free diet.

The mean age of the avocado group patients was 49.7 years, while the control group was 50.1 years. Tables 1-2 contain baseline characteristics of the included studies related to avocado consumption and its effects.

**Table 1:** Baseline characteristics of the included studies. AAD-LFD, Average American diet-low-fat diet; NR, not reported

Study ID	Country	Study design	Avocado (g/day)	Control diet	Sample size		Age (years)		Male (N)		Female (N)		BMI (kg/m2)	
					Avocado	Control	Avocado	Control	Avocado	Control	Avocado	Control	Avocado	Control
Pieterse et al. (2005) [24]	South Africa	Randomized clinical trial	200	Energy restricted diet	28	27	40.8 ± 8.9		NR	NR	NR	NR	31.9 ± 3.9	
Scott et al. (2017) [25]	United States	Randomized controlled trial		One potato or one cup of chickpeas	20	20	63.3 ± 11	62.5 ± 9.2	6	9	14	11	24.1 ± 3.1	24.2 ± 2.4
Wang et al. (2015) [1]	Australia	Randomized, crossover, controlled feeding trial	One Hass avocado	AAD-LFD	45		45 ± 13.3		NR		NR		28.2 ± 2.4	
Zhang et al. (2022) [26]	United States	Randomized clinical trial	One Hass avocado	Low-fat diet	49	45	40.6 ± 11.8	42.7 ± 12.5	21	18	28	26	92.2 ± 14.7	32.8 ± 3.88
Colquhoun et al. (1992) [27]	Australia	Randomized clinical trial	200-500	Low-fat diet	15	15	49	49	0		15		66.8	
Lichtenstein et al. (2022) [28]	United States	Randomized, controlled, parallel-arm, unblinded trial	NR	Habitual diet	505	503	50.1 ± 14.3	50.4 ± 13.8	149	129	356	374	32.9 ± 5.3	33.2 ± 5.6
Alvizouri-Muñoz et al. (1992) [29]	Italy	Randomized clinical trial	NR	Habitual diet	21	21	NR	NR	NR	NR	NR	NR	NR	NR

**Table 2:** Age, statin status, and dietary breakdown. AAD, average American diet; CHO, carbohydrate

Study ID	Age (years)	Statin use	Socioeconomic status	Control diet
Pieterse et al. (2005) [24]	21 to 57	Six subjects (four in the experimental group and two in the control group) used hypolipidemic agents (statins).	Not reported	Mixed dietary fats such as margarine or oil. (Other specifics of the control diet were not given.)
Scott et al. (2017) [25]	>=50	individuals taking medications interfering with fat absorption (e.g., bile acid sequestrants, ezetimibe) were excluded.	Not reported	One medium potato or one cup of chickpeas a day.
Wang et al (2015) [1]	21-70	All participants were not taking lipid‐lowering medications or supplements.	Not reported	The control diet was described as AAD: 34% fat, 51% CHO, and 16% protein.
Zhang et al. (2022) [26]	25-65	Individuals who used medications that would interfere with the study's outcomes, like lipid-lowering medications, were excluded.	Not reported	Participants randomly allocated to the control arm were given control foods in different combinations to match the energy level of one avocado per day as closely as possible. Foods such as mini bagels, pierogis, fruit juice, waffle, instant oatmeal, and others were provided.
Colquhoun et al. (1992) [27]	37 and 58	The study was silent on statins or lipid-lowering agents.	Not reported	High-complex-carbohydrate diet (AHA-III) on blood lipid concentrations. (Specifics of this diet were not reported.)
Lichtenstein et al. (2022) [28]	>25	Statin intake was not reported in the inclusion or exclusion criteria.	Not reported	The control diet was described as a Habitual diet (with no specifics).
Madrigal 2008 [29]	Did not report age range	Was silent on statin.	Not reported	The control diets were labeled as Rich monosaturated, free monosaturated, and Low monosaturated fats (Without specific mention of types of traditional food names). The first and third diets were designed to simulate a usual diet, and volunteers carried on their normal activities during the trial.

Table 3 provides information on various parameters related to the studies that investigated the effects of avocado consumption on cholesterol levels, blood pressure, and glucose levels. 

**Table 3:** Table displaying cholesterol levels, blood pressure, and glucose measurements in avocado and control groups from various research papers. NR, not reported

Study ID	Total cholesterol (mg/dL)		LDL cholesterol (mg/dL)		HDL cholesterol (mg/dL)		Triglyceride (mg/dL)		Systolic blood pressure		Diastolic blood pressure		Glucose (mg/dL)	
	Avocado	Control	Avocado	Control	Avocado	Control	Avocado	Control	Avocado	Control	Avocado	Control	Avocado	Control
Pieterse et al. (2005) [24]	5.19 ± 1.17		3.45 ± 1.12		1.1 ± 0.31		1.73 ± 2.03		120 ± 15.6		71 ± 8.2		NR	
Scott et al. (2017) [25]	NR		NR		NR		NR		NR		NR		NR	
Wang et al. (2015) [1]	199.9 ± 32.4		128.1 ± 26.1		48.7 ± 12.1		114.0 ± 39.6		117.2± 10.4		79.2 ± 7.4		92.2 ± 8.0	
Zhang et al. (2022) [26]	NR		NR		NR		NR		NR		NR		106 ± 9.73	104 ± 11.1
Colquhoun et al. (1992) [27]	NR		NR		NR	NR	NR	NR	NR		NR		NR	
Lichtenstein et al. (2022) [28]	185 ± 40	190 ± 39	110 ± 34	114 ± 34	55 ± 13 WOMEN 44 ± 9 MEN	56 ± 13 WOMEN 45 ± 10 MEN	110 ± 49	107 ± 54	123 ± 16	123 ± 16	77 ± 10	76 ± 11	107 ± 29	107 ± 30
Alvizouri-Muñoz et al. (1992) [29]	212.68 ± 42.5		119.9 ± 11.2		30.9 ± 7.3		336 ± 256		NR	NR	NR	NR	189 ± 70.2	

Results of risk-of-bias assessment

**Figure 2:**
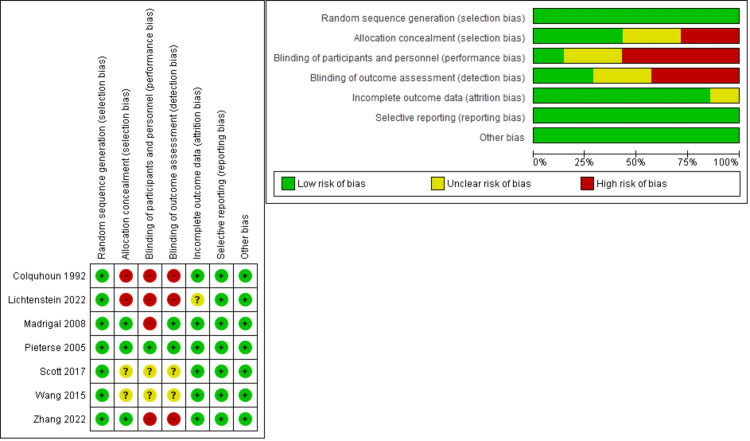
Risk-of-bias table. Colquhoun et al. (1992) [27], Lichtenstein et al. (2022) [28], Alvizouri-Muñoz et al. (1992) [29], Pieterse et al. (2005) [24], Scott et al. (2017) [25], Wang et al. (2015) [1], Zhang et al. (2022) [26]

According to Cochrane's tools, the risk-of-bias evaluation of RCTs indicated an overall low risk of bias. Regarding randomization, all the involved studies were at low risk. Regarding allocation concealment, three studies [23,26,29] were at low risk, and two studies [27,28] were at high risk, but the rest of the studies [24,25] did not report enough data. All the studies were not blinded to the participants and personnel except two studies [24,25] that did not report enough data, and Pieterse et al. [23] were blinded. Three studies [26-28] were not blinded to outcome assessment, two studies [23,29] were blinded, and two studies [24,25] did not report enough data. A summary of the risk of bias in involved trials is demonstrated in Figure 2.

Results

Analysis of the Outcomes

According to the control group's diet type, we conducted a subgroup analysis and divided the studies into two subgroups: the habitual diet (HD) group and the low-fat (LF) diet group.

**Figure 3:**
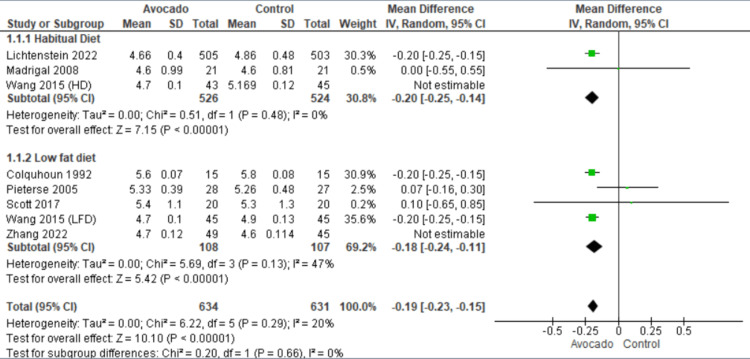
Analysis of studies that reported total cholesterol. Colquhoun et al. (1992) [27], Lichtenstein et al. (2022) [28], Alvizouri-Muñoz et al. (1992) [29], Pieterse et al. (2005) [24], Scott et al. (2017) [25], Wang et al. (2015) [1], Zhang et al. (2022) [26] IV, intravenous; SD, standard deviation; CI, confidence interval

Total cholesterol: Seven studies [23-29] reported the levels of TC. In the HD subgroup, the avocado group was accompanied by lower levels of TC than the control group (MD = -0.20 [-0.25, -0.14], *P* = 0.0001). The data were heterogeneous and homogenous after excluding Wang et al. [1] from the leave-one-out method (*I*^2^ = 0%, *P* = 0.48). In the LF diet subgroup, the avocado group was accompanied by lower levels of TC than the control group (MD = -0.18 [-0.24, -0.11], *P* = 0.0001). The data were heterogeneous and became homogenous after excluding Zhang et al. [26] by the leave-one-out method (*I*^2^ = 47%, *P* = 0.13) (Figure 3).

**Figure 4:**
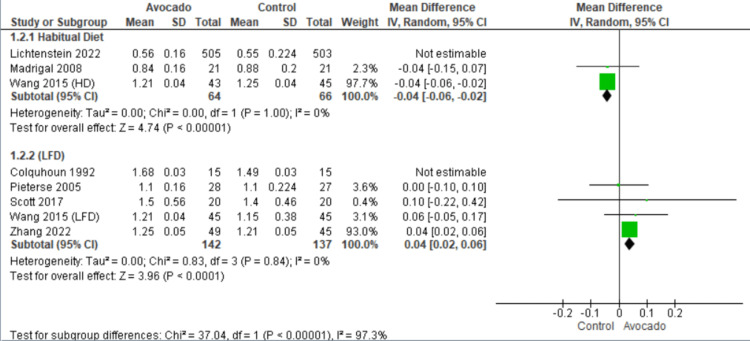
Analysis of studies that reported HDL. Colquhoun et al. (1992) [27], Lichtenstein et al. (2022) [28], Madrigal et al. (2008) [29], Pieterse et al. (2005) [24], Scott et al. (2017) [25], Wang et al. (2015) [1], Zhang et al. (2022) [26] IV, intravenous; SD, standard deviation; CI, confidence interval; HDL, high-density lipoprotein

High-density lipoprotein: The levels of HDL were reported by seven studies [23-29]. In the HD subgroup, we found that the control group was associated with higher levels of HDL than the avocado group (MD = -0.04 [-0.06, -0.02], *P* = 0.0001). The data were heterogeneous and became homogenous after excluding Lichtenstein et al. [28] by the leave-one-out method (*I*^2^ = 0%, *P* = 1.0). In the LF diet subgroup, the avocado group showed higher levels of HDL than the control group (MD = -0.04 [-0.06, -0.02], *P* = 0.0001). The data were heterogeneous and became homogenous after excluding Colquhoun et al. [27] by the leave-one-out method (*I*^2^ = 0%, *P* = 0.84) (Figure 4).

**Figure 5:**
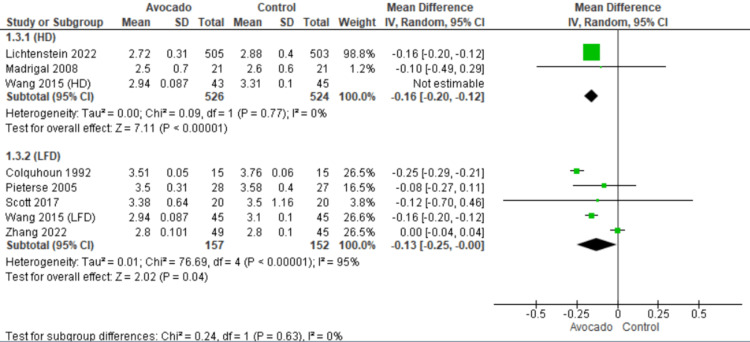
Analysis of studies that reported LDL. Colquhoun et al. (1992) [27], Lichtenstein et al. (2022) [28], Madrigal et al. (2008) [29], Pieterse et al. (2005) [24], Scott et al. (2017) [25], Wang et al. (2015) [1], Zhang et al. (2022) [26] IV, intravenous; SD, standard deviation; CI, confidence interval; LDL, low-density lipoprotein

Low-density lipoprotein: All the included studies [23-29] reported LDL levels. In both subgroups, HD and LF subgroups, the LDL levels were lower in the avocado group than in the control group (MD = -0.16 [-0.20, -0.12], *P* = 0.0001) and (MD = -0.13 [-0.25, -0.00], *P* = 0.04), respectively. In the HD subgroup, the data were heterogeneous and became homogenous after excluding Wang et al. [1] by the leave-one-out method (I2 = 0%, *P* = 0.77). In the LF subgroup, the data showed heterogeneity (*I*^2^ = 95%, *P* = 0.0001) (Figure 5).

**Figure 6:**
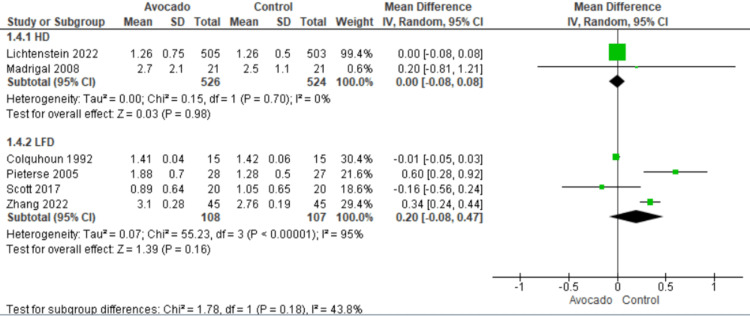
Analysis of studies that reported TG. Colquhoun et al. (1992) [27], Lichtenstein et al. (2022) [28], Alvizouri-Muñoz et al. (1992) [29], Pieterse et al. (2005) [24], Scott et al. (2017) [25], Wang et al. (2015) [1], Zhang et al. (2022) [26]. IV, intravenous; SD, standard deviation; CI, confidence interval; TG, triglyceride

Triglyceride: Six studies [23,24,26-29] reported the levels of TG. We found that the TG levels were similar in both groups regarding both subgroups: HD subgroup (MD = 0.00 [-0.08, 0.08], *P* = 0.98) and LF subgroup (MD = 0.20 [-0.08, 0.47], *P* = 0.16). The data were homogenous in the HD subgroup (*I*^2^ = 0%, *P* = 0.7) and heterogeneous in the LF subgroup (*I*^2^ = 95%, *P* = 0.0001) (Figure 6).

**Figure 7:**
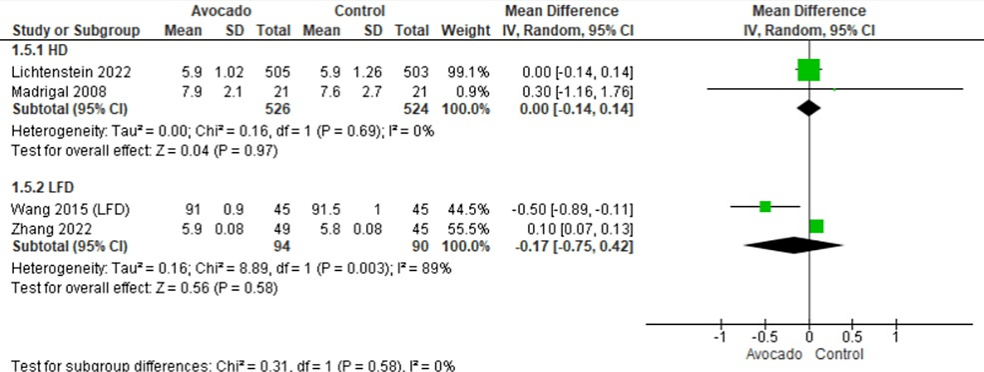
Analysis of studies that reported FBG. Colquhoun et al. (1992) [27], Lichtenstein et al. (2022) [28], Alvizouri-Muñoz et al. (1992) [29], Pieterse et al. (2005) [24], Scott et al. (2017) [25], Wang et al. (2015) [1], Zhang et al. (2022) [26]. IV, intravenous; SD, standard deviation; CI, confidence interval, FBG, fasting blood glucose

Fasting blood glucose: The outcome was reported by four studies [25,26,28,29]. We found that the FBG levels were similar in both groups regarding both subgroups: HD subgroup (MD = 0.00 [-0.14, 0.14], *P* = 0.97) and LF subgroup (MD = - 0.17 [-0.75, 0.42], *P* = 0.58). The data were homogenous in the HD subgroup (*I*^2^ = 0%, *P* = 0.69) and heterogeneous in the LF subgroup (*I*^2^ = 89%, *P* = 0.003) (Figure 7).

**Figure 8:**
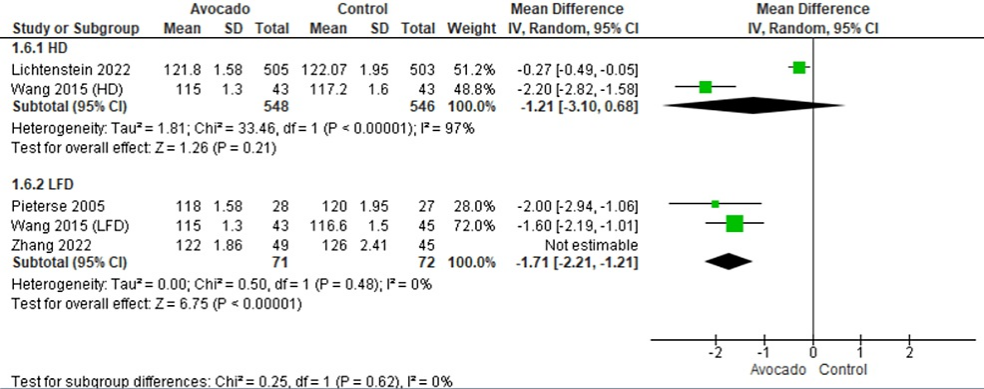
Analysis of studies that reported systolic blood pressure. Colquhoun et al. (1992) [27], Lichtenstein et al. (2022) [28], Alvizouri-Muñoz et al. (1992) [29], Pieterse et al. (2005) [24], Scott et al. (2017) [25], Wang et al. (2015) [1], Zhang et al. (2022) [26]. IV, intravenous; SD, standard deviation; CI, confidence interval

Systolic blood pressure: SBP was reported by four studies [23,25,26,28]. Regarding the HD subgroup, the SBP was the same in both the avocado and the control group (MD = -1.21 [-3.10, 0.68], *P* = 0.21). The data showed heterogeneity (*I*^2^ = 97%, *P* = 0.0001). Regarding the LF subgroup, the SBP decreased with the avocado group than the control group (MD = -1.71 [-2.21, -1.21], *P* = 0.001). The data were heterogeneous and became homogeneous after excluding Zhang et al. [26] by the leave-one-out method (*I*^2^ = 0%, *P* = 0.48) (Figure 8).

**Figure 9:**
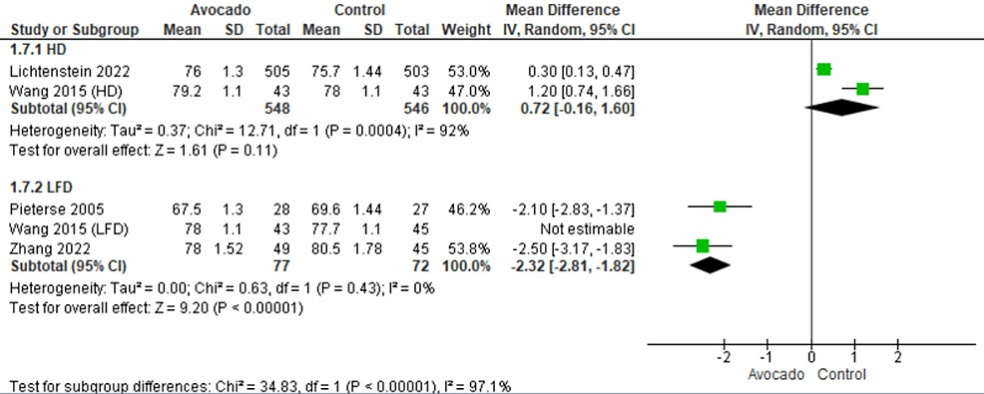
Analysis of studies that reported diastolic blood pressure. Colquhoun et al. (1992) [27], Lichtenstein et al. (2022) [28], Alvizouri-Muñoz et al. (1992) [29], Pieterse et al. (2005) [24], Scott et al. (2017) [25], Wang et al. (2015) [1], Zhang et al. (2022) [26]. IV, intravenous; SD, standard deviation; CI, confidence interval

Diastolic blood pressure: DBP was reported by four studies [23,25,26,28]. Regarding the HD subgroup, the DBP was the same in both the avocado and the control group (MD = 0.72 [-0.16, 1.60], *P* = 0.11). The data showed heterogeneity (*I*^2^ = 92%, *P* = 0.0004). Regarding the LF subgroup, the DBP decreased with the avocado group than the control group (MD = -2.32 [-2.81, -1.82], *P* = 0.0001). The data were heterogeneous and became homogeneous after excluding Wang et al. [1] by the leave-one-out method (*I*^2^ = 0%, *P* = 0.43) (Figure 9).

## Review

In this meta-analysis, we investigated the efficacy of the avocado-rich diet in reducing the risk of CVDs compared with HD and LF diets. Our analysis showed that the avocado group was accompanied by lower levels of TC than the HD and LF diets. Concerning HDL, we found that HD was associated with higher levels of HDL compared with the avocado diet. On the other hand, populations in the LF diet group had higher levels of HDL compared with the group following the avocado-rich diet. Regarding the LDL levels, the avocado diet was associated with lower levels of LDL. We also found that TG levels were similar in different diet groups.

A randomized trial by Wang et al. [1] found that consuming one avocado per day for six weeks resulted in lower levels of circulating oxLDL and increased plasma lutein concentrations. This effect was observed compared to a typical Western diet, a moderate-fat diet with matched macronutrients and fatty acids, and a high-carbohydrate, low-fat diet. The benefits were attributed to the bioactive compounds present in avocados, which go beyond their fatty acids. The reduction in small LDL particles may have contributed to lowering oxLDL on the avocado diet. Furthermore, increasing the intake of fruits, vegetables, and whole grains may protect against the oxidation of atherogenic lipoproteins even on a high-carbohydrate, low-fat diet that increases small, dense LDL (sdLDL) particles [30].

Pacheco et al. [31] conducted a comprehensive study on American men and women, revealing that consuming avocados regularly can significantly reduce the risk of developing CVDs and coronary heart diseases. However, no significant association was found between avocado intake and total or ischemic stroke. The study also suggested replacing unhealthy food items such as margarine, butter, processed meats, cheese, and eggs with avocados can lower the incidence of CVD. This study provides further evidence that incorporating plant-based unsaturated fats in our diet can improve its quality and play a crucial role in preventing CVD in the general population.

Wang et al. [1] found that consuming one Hass avocado per day can have positive effects on reducing LDL and other emerging risk factors for CVD beyond just its fatty acid content. This study also shows that a moderate-fat diet low in saturated fat and high in monounsaturated fat from avocados can lead to greater reductions in various cholesterol markers compared to a high-monounsaturated fat diet with similar macronutrient and fatty acid profiles. Therefore, incorporating a food source rich in monounsaturated fats and bioactive compounds can provide additional cardiovascular benefits compared to a low-saturated-fat diet with matched levels of monounsaturated fats. They also discovered that individuals who followed a high-fat diet had higher HDL levels than those who followed an avocado diet. Conversely, those in the low-fat diet group had higher HDL levels than the avocado diet group, which is similar to our results.

In terms of FBG levels, our analysis showed that different diet groups were not associated with significant changes in glucose levels. Lichtenstein et al. [28] reported no notable variations in the FBG between the HD and avocado diet after six months of providing one avocado daily. In addition, Wang et al. [1] showed that fasting blood sugar did not differ between HD, LF diet, avocado diet, and moderate-fat diet.

Normal ranges of high blood pressure have evolved over the years and diet composition may play an important role [32-33]. We studied the effect of an avocado-rich diet on blood pressure and found that the SBP was the same in both the avocado and HD groups. However, the SBP decreased with the avocado group than with the LF diet group. We noticed similar results concerning the DBP. The DBP remained consistent between the avocado and HD groups, but the avocado group showed a more significant decrease in DBP than the LF diet group.

Cheng et al. [32] suggested that consuming tomato products and taking lycopene supplements can benefit cardiovascular risk factors such as blood pressure, blood lipids, and endothelial function. This supports the idea of developing personalized nutritional plans that incorporate tomatoes to combat CVD. Lichtenstein et al. [28] Reported that SBP and DBP did not differ significantly between diet groups.

Pieterse et al. [24] showed that neither avocado (MUFA) nor weight loss had any impact on serum lipid levels, plasma fibrinogen, arterial compliance, SBP, or DBP. However, the limited sample size may have obscured any potential effects on these variables. A prior meta-analysis conducted by Mahmassani et al. examined the impact of avocado consumption on CVDs. The findings indicated that incorporating avocados into one's diet may enhance HDL cholesterol levels. Nonetheless, avocado intake did not significantly influence serum TC, LDL cholesterol, or TG values [34].

Study limitations

One of the main limitations of our meta-analysis is the number of studies and the significant heterogeneity in some outcomes. However, we managed to solve the heterogeneity through the leave-one-out method. The analysis showed significant differences among studies due to variations in factors such as the types of participants, their dietary habits, the control groups used, and the length of the intervention. We performed a subgroup analysis according to the control group, which provided more homogenous results.

Another limitation is that the studies included in the analysis had small sample sizes and significant variations in the results. Therefore, this finding should be interpreted with caution, and further long-term trials are needed to fully understand the impact of avocado consumption on blood lipid levels and other risk factors for CVD.

## Conclusions

After analyzing the data, we discovered that individuals in the avocado group had lower levels of TC than those in the HD and low-fat diet groups. The HD group had higher levels of HDL than the avocado group, while the low-fat diet group had higher levels of HDL than the avocado group. The avocado diet was associated with lower LDL levels. The avocado diet did not affect the TG and fasting glucose levels with minimal effect on SBP values. While our analysis revealed significant differences in cholesterol levels among individuals in the avocado, HD, and low-fat diet groups, further research is needed to explore several aspects. First, future studies should investigate the long-term effects of the avocado diet on cholesterol levels to determine if the observed differences persist over time. Additionally, it would be valuable to examine the potential mechanisms by which avocados may impact cholesterol metabolism, shedding light on the underlying biological processes involved. Furthermore, considering the differing effects on HDL levels between the avocado group and the HD and low-fat diet groups, future research could focus on identifying the specific dietary components responsible for these variations. Moreover, it would be beneficial to investigate the impact of the avocado diet on other cardiovascular risks factors, such as stroke, myocardial infarction, and other vasculo-cardiac comorbid outcomes of cardiovascular disease to gain a more comprehensive understanding of its overall effects on cardiometabolic health.
