# Correction: Effect of Avocado Consumption on Risk Factors of Cardiovascular Diseases: A Systematic Review and Meta-Analysis

**DOI:** 10.7759/cureus.c160

**Published:** 2024-02-13

**Authors:** Okelue E Okobi, Victor A Odoma, Omolola Okunromade, Olusayo Louise-Oluwasanmi, Blessing Itua, Chinonso Ndubuisi, Omosefe E Ogbeifun, Bright C Nwatamole, Thomas A Elimihele, Joy O Adekunle, Akeem A Adekunle, Chinedum B Obi, Endurance O Evbayekha

**Affiliations:** 1 Family Medicine, Larkin Community Hospital, Miami, USA; 2 Family Medicine, Lakeside Medical Center, Belle Glade, USA; 3 Cardiology/Oncology, Indiana University (IU) Health Bloomington Hospital, Bloomington, USA; 4 Public Health, Jiann-Ping Hsu College of Public Health, Georgia Southern University, Statesboro, USA; 5 Genetics, Howard University College of Medicine, Washington, USA; 6 Internal Medicine, Annotto Bay Hospital, St. Mary, JAM; 7 Family Medicine, Humboldt Park Health, Chicago, USA; 8 Public Health, University of West Florida, Florida, USA; 9 Cardiology, Calderdale and Huddersfield NHS Foundation Trust, Huddersfield , GBR; 10 Clinical Research, Norris Comprehensive Cancer Center, University of Southern California, Los Angeles, USA; 11 Internal Medicine, Lagos State Health Service (LHSC), Lagos, NGA; 12 Internal Medicine, College of Medicine, University of Lagos, Lagos, NGA; 13 Internal Medicine, Imo State University, Owerri, NGA; 14 Internal Medicine, St. Luke's Hospital, Chesterfield, USA

This article has been corrected to accurately reflect the HDL-C concentrations in Table 3.

Previously the values for the HDL cholesterol (mg/dL) avocado group and HDL cholesterol (mg/dL) control group were incorrectly stated as 146.9 ± 38.6 and 143 ± 38.6, respectively. These values have been corrected to 55 ± 13 for the avocado group (women) and 44 ± 9 for the avocado group (men). Control group (women) is now 56 ± 13 and the control group (men) is 45 ± 10.

The authors and journal regret that these errors were not identified and addressed prior to publication.

 

